# *catena*-Poly[[bis­[aqua­(1,10-phenanthroline)lead(II)]-bis­(μ_3_-2-hy­droxy-5-sulfonato­benzoato)] acetic acid monosolvate]

**DOI:** 10.1107/S160053681202421X

**Published:** 2012-06-13

**Authors:** Yuan-Zheng Cheng, Wei-Wei Shi, Xue-Dong Wang, Shu-E Deng, Li-Ping Zhang

**Affiliations:** a Department of Chemistry, Weifang Medical University, Weifang 261053, People’s Republic of China

## Abstract

In the title compound, [Pb_2_(C_7_H_4_O_6_S)_2_(C_12_H_8_N_2_)_2_(H_2_O)_2_]·CH_3_COOH, the seven-coordinate Pb^II^ atom is chelated by two N atoms of one 1,10-phenanthroline ligand, four O atoms from three 5-sulfosalicylate dianions and one water O atom. Each dianion serves as a bridging ligand, connecting adjacent Pb^II^ atoms into a centrosymmetric polymeric chain extending parallel to [001]. There are π–π inter­actions between the aromatic systems of neighbouring dianions, with plane-to-plane distances of 3.371 (2) Å, and between phenanthroline ligands, with a centroid-to-centroid distance of 3.484 (2) Å. O—H⋯O hydrogen bonding additionally stabilizes the crystal packing. The acetic acid mol­ecules are incorporated in the voids of this arrangement. They exhibit half-occupancy due to disorder around a centre of inversion.

## Related literature

For background to 5-sulfosalicylic acid and its metal complexes, see: Chen *et al.* (2003 ▶); Du *et al.* (2006 ▶); Fan & Zhu (2005*a* ▶,*b* ▶, 2006 ▶); Li *et al.* (2004 ▶).
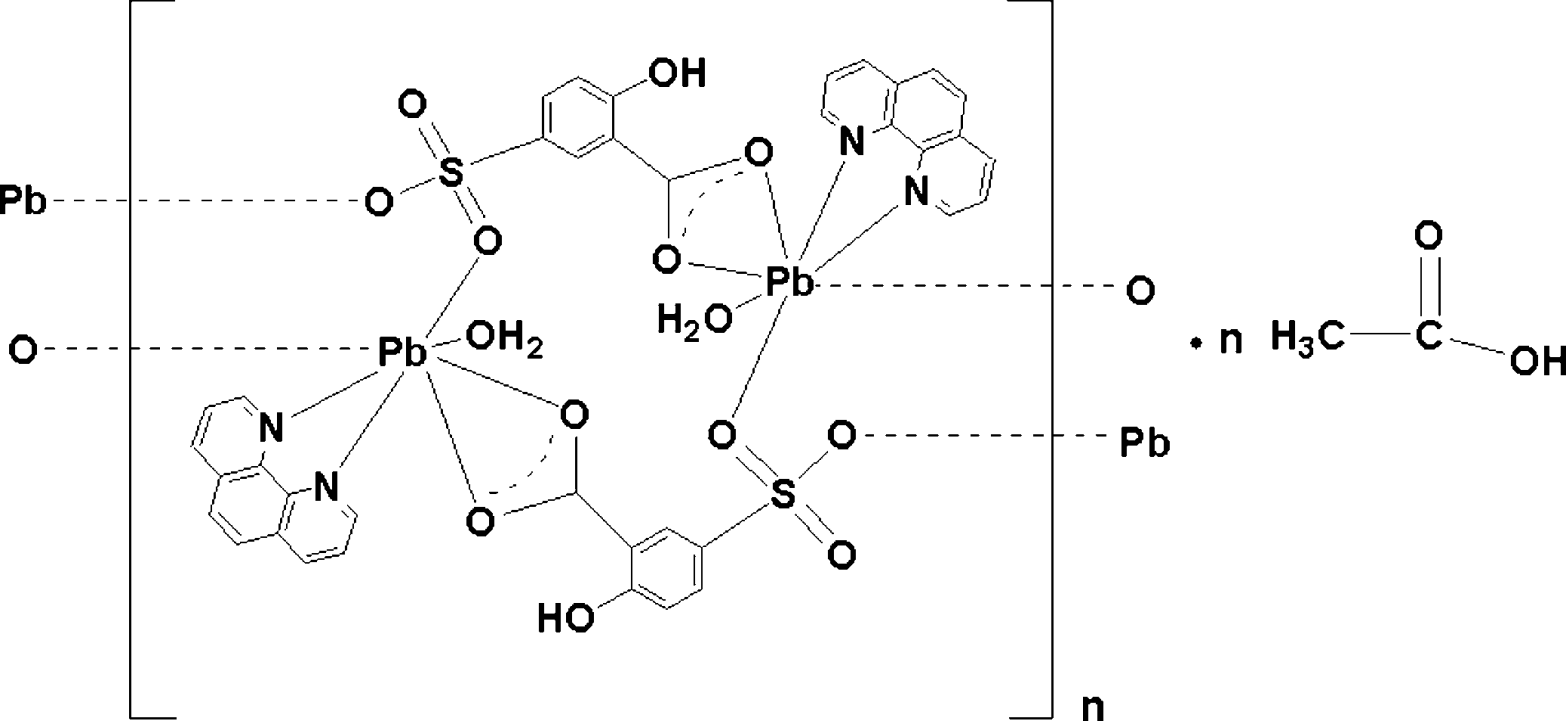


## Experimental

### 

#### Crystal data

[Pb_2_(C_7_H_4_O_6_S)_2_(C_12_H_8_N_2_)_2_(H_2_O)_2_]·C_2_H_4_O_2_
*M* *_r_* = 1303.24
Triclinic, 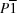

*a* = 9.7372 (7) Å
*b* = 10.1619 (5) Å
*c* = 11.6637 (5) Å
α = 74.171 (4)°
β = 74.379 (5)°
γ = 79.748 (5)°
*V* = 1062.50 (11) Å^3^
*Z* = 1
Mo *K*α radiation
μ = 8.09 mm^−1^
*T* = 293 K
0.15 × 0.13 × 0.12 mm

#### Data collection

Oxford Xcalibur CCD diffractometer
Absorption correction: multi-scan (*CrysAlis PRO*; Oxford Diffraction, 2011 ▶) *T*_min_ = 0.309, *T*_max_ = 0.379
6374 measured reflections
3745 independent reflections
3438 reflections with *I* > 2σ(*I*)
*R*_int_ = 0.036

#### Refinement

*R*[*F* ^2^ > 2σ(*F* ^2^)] = 0.027
*wR*(*F* ^2^) = 0.064
*S* = 1.05
3745 reflections
310 parameters
28 restraints
H-atom parameters constrained
Δρ_max_ = 1.83 e Å^−3^
Δρ_min_ = −0.84 e Å^−3^

### 

Data collection: *CrysAlis PRO* (Oxford Diffraction, 2011 ▶); cell refinement: *CrysAlis PRO*; data reduction: *CrysAlis PRO*; program(s) used to solve structure: *SHELXS97* (Sheldrick, 2008 ▶); program(s) used to refine structure: *SHELXL97* (Sheldrick, 2008 ▶); molecular graphics: *ORTEP-3 for Windows* (Farrugia, 1997 ▶) and *DIAMOND* (Brandenburg, 2006 ▶); software used to prepare material for publication: *publCIF* (Westrip, 2010 ▶).

## Supplementary Material

Crystal structure: contains datablock(s) I, global. DOI: 10.1107/S160053681202421X/wm2635sup1.cif

Structure factors: contains datablock(s) I. DOI: 10.1107/S160053681202421X/wm2635Isup2.hkl

Additional supplementary materials:  crystallographic information; 3D view; checkCIF report

## Figures and Tables

**Table 1:** Selected bond lengths (Å)

Pb1—O6	2.472 (3)
Pb1—N2	2.473 (3)
Pb1—N1	2.488 (3)
Pb1—O5	2.745 (3)
Pb1—O3^i^	2.757 (3)
Pb1—O7	2.835 (4)
Pb1—O1^ii^	2.792 (1)

Symmetry codes: (i) 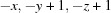; (ii) 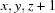.

**Table 2:** Hydrogen-bond geometry (Å, °)

*D*—H⋯*A*	*D*—H	H⋯*A*	*D*⋯*A*	*D*—H⋯*A*
O4—H4⋯O6	0.82	1.83	2.522 (4)	141
O7—H7*A*⋯O2^iii^	0.85	2.11	2.959 (5)	172

Symmetry code: (iii) 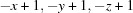.

**Fig. 1.**
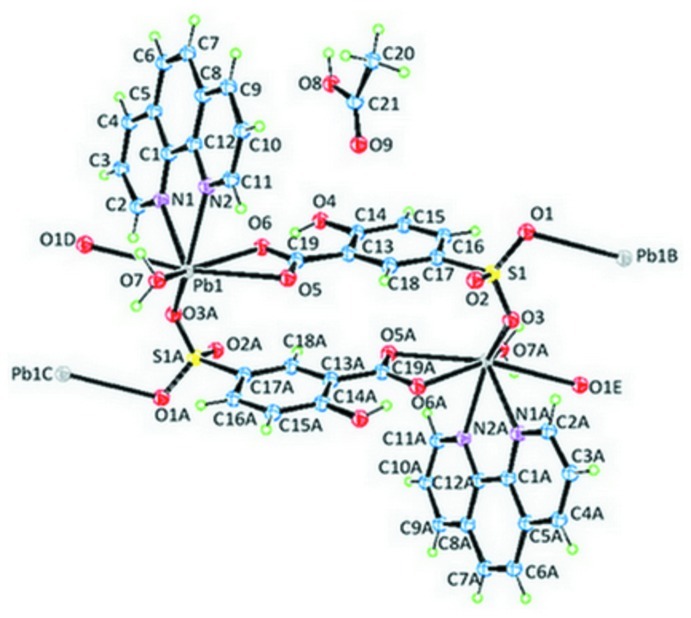
The molecular structure of the title compound with displacement ellipsoids drawn at the 30% probability level. [Symmetry codes: (A) -x, -y + 1, -z + 1; (B) x, y, z + 1]. Only one orientation of the disordered acetic acid molecule is shown.

**Fig. 2.**
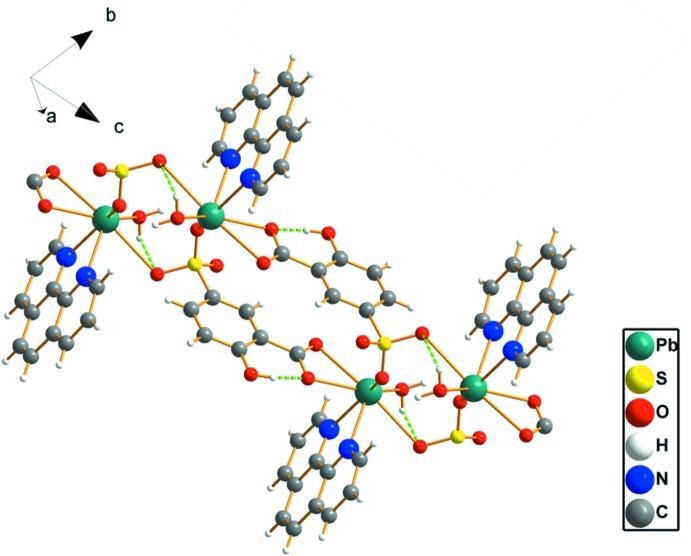
Part of the crystal structure of the title compound, showing O—H···O hydrogen bonds as green dashed lines.

**Fig. 3.**
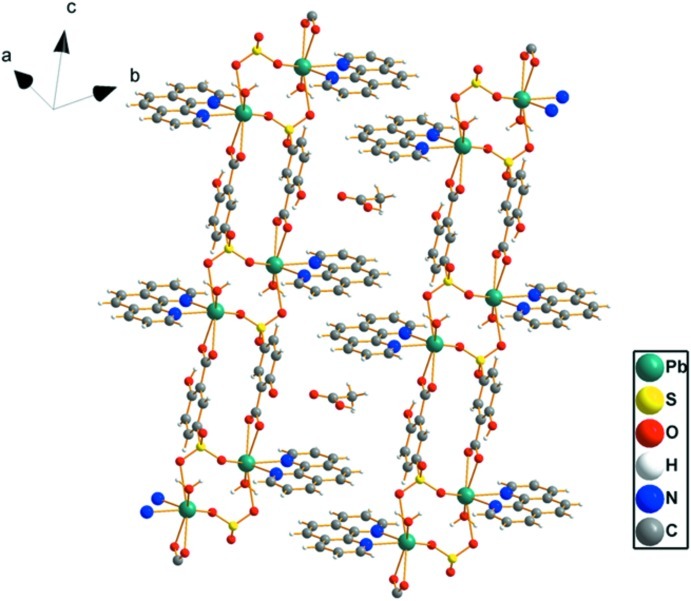
The π–π stacking of the 1,10-phenanthroline units in the title compound. Only one orientation of the disordered acetic acid molecule is shown.

## supplementary crystallographic information

### Comment

5-Sulfosalicylic acid (H_3_ssal) has three functional groups, *viz* -SO_3_H, -COOH and -OH, and can form three kind of ions (H_2_ssal^-^, Hssal^2-^ and ssal^3-^) (Fan & Zhu, 2006). Hssal^2-^ possesses the ability to bridge and chelate metal atoms using the carboxylate O atoms and the sulfonate O atoms (Chen *et al.*, 2003). A few complexes containing Hssal^2-^ and phen ligands have been reported (Chen *et al.*, 2003; Li *et al.*, 2004; Fan & Zhu, 2005*a*,*b*, 2006; Du *et al.*, 2006). Among the aforementioned complexes, there is a polymer (Fan & Zhu, 2006), which contains Pb^II^ atoms and *N*,*N*-dimethylformamide ligands. In this paper, we report a new polymeric Pb^II^ complex which contains water ligands instead of *N*,*N*-dimethylformamide ligands and acetic acid solvent molecules in the crystal lattice.

The Pb^II^ atom is seven-coordinated by two N atoms of one phenanthroline ligand, four O atoms from three Hssal^2-^ ligands, and one O atom from one H_2_O molecule (Fig. 1). The Pb—O distances range from 2.472 (3) Å to 2.835 (4) Å, and the Pb—N distances from 2.473 (3) Å to 2.488 (3) Å (Table 1). The distances of Pb···Pb separated by the Hssal^2-^ ligand are 8.9162 (4) Å and 11.6637 (5) Å, while the Pb···Pb separation by sulfonate is 5.0722 (3) Å.

In the crystal, there is an intramolecular O4—H4···O6 hydrogen bond in the Hssal^2-^ ligand. In addition, there are intermolecular O—H···O hydrogen bonds linking water molecular and Hssal^2-^ ligands (Table 2 and Fig. 2). Aromatic π–π stacking occurs between neighbouring phenanthroline ligands (Fig. 3). The centroid–centroid distance between *Cg*3 (C1/C5–C8/C12) and *Cg*3 (C1/C5–C8/C12) [symmetry code: 1 - *x*, -*y*, 2 - *z*] is 3.484 (2) Å. π–π interactions between the aromatic systems of neighbouring dianions with plane-to-plane distances of 3.371 (2) Å also occur.

In the voids of this arrangement acetic acid molecules are incorporated. They exhibit half-occupancy due to disorder around a centre of inversion.

### Experimental

A mixture of Pb(CH_3_COO)_2_.3H_2_O (0.5 mmol, 0.1897 g) and 1.10-phenanthroline (0.5 mmol, 0.0991 g) in a solution of dimethylacetylamide (DMAC; 20 ml) was stirred for 20 min. Then 5-sulfosalicylic acid dihydrate (0.5 mmol, 0.1271 g) and NaOH (0.5 mmol, 0.0200 g) were dissolved in water (20 ml), which was added dropwise into the previous solution under stirring. The mixed solution was stirred for 1 h and filtered. The resulting solution was set aside for evaporation at room temperature for 11 d, and colorless block-shaped single crystals were obtained.

### Refinement

The H atoms were placed at calculated positions and were allowed to ride on their parent atoms,with C—H = 0.93 (aromatic C—H), 0.96 (methyl) and O—H = 0.82 (hydroxyl), 0.85 (water) Å, with *U*_iso_(H) = 1.2*U*_eq_(aromatic C), *U*_iso_(H)= 1.5*U*_eq_(methyl C) and *U*_iso_(H) = 1.5*U*_eq_(O). The acetic acid molecule is disordered around an inversion centre and was modelled with half-occupancy.

### Crystal data

**Table:**

[Pb_2_(C_7_H_4_O_6_S)_2_(C_12_H_8_N_2_)_2_(H_2_O)_2_]·C_2_H_4_O_2_	*Z* = 1
*M**_r_* = 1303.24	*F*(000) = 624
Triclinic, *P*1	*D*_x_ = 2.037 Mg m^−^^3^
Hall symbol: -P 1	Mo *K*α radiation, λ = 0.71073 Å
*a* = 9.7372 (7) Å	Cell parameters from 4152 reflections
*b* = 10.1619 (5) Å	θ = 2.5–28.4°
*c* = 11.6637 (5) Å	µ = 8.09 mm^−^^1^
α = 74.171 (4)°	*T* = 293 K
β = 74.379 (5)°	Block, colourless
γ = 79.748 (5)°	0.15 × 0.13 × 0.12 mm
*V* = 1062.50 (11) Å^3^	

### Data collection

**Table:**

Oxford Xcalibur CCD diffractometer	3745 independent reflections
Radiation source: fine-focus sealed tube	3438 reflections with *I* > 2σ(*I*)
Graphite monochromator	*R*_int_ = 0.036
Detector resolution: 16.2563 pixels mm^-1^	θ_max_ = 25.0°, θ_min_ = 3.1°
ω scans	*h* = −11→9
Absorption correction: multi-scan (*CrysAlis PRO*; Oxford Diffraction, 2011)	*k* = −12→12
*T*_min_ = 0.309, *T*_max_ = 0.379	*l* = −13→13
6374 measured reflections	

### Refinement

**Table:**

Refinement on *F*^2^	Primary atom site location: structure-invariant direct methods
Least-squares matrix: full	Secondary atom site location: difference Fourier map
*R*[*F*^2^ > 2σ(*F*^2^)] = 0.027	Hydrogen site location: inferred from neighbouring sites
*wR*(*F*^2^) = 0.064	H-atom parameters constrained
*S* = 1.05	*w* = 1/[σ^2^(*F*_o_^2^) + (0.0245*P*)^2^ + 1.8039*P*] where *P* = (*F*_o_^2^ + 2*F*_c_^2^)/3
3745 reflections	(Δ/σ)_max_ = 0.002
310 parameters	Δρ_max_ = 1.83 e Å^−^^3^
28 restraints	Δρ_min_ = −0.84 e Å^−^^3^

### Special details

**Table:**

Geometry. All e.s.d.'s (except the e.s.d. in the dihedral angle between two l.s. planes) are estimated using the full covariance matrix. The cell e.s.d.'s are taken into account individually in the estimation of e.s.d.'s in distances, angles and torsion angles; correlations between e.s.d.'s in cell parameters are only used when they are defined by crystal symmetry. An approximate (isotropic) treatment of cell e.s.d.'s is used for estimating e.s.d.'s involving l.s. planes.
Refinement. Refinement of *F*^2^ against ALL reflections. The weighted *R*-factor *wR* and goodness of fit *S* are based on *F*^2^, conventional *R*-factors *R* are based on *F*, with *F* set to zero for negative *F*^2^. The threshold expression of *F*^2^ > σ(*F*^2^) is used only for calculating *R*-factors(gt) *etc*. and is not relevant to the choice of reflections for refinement. *R*-factors based on *F*^2^ are statistically about twice as large as those based on *F*, and *R*-factors based on ALL data will be even larger.

### Fractional atomic coordinates and isotropic or equivalent isotropic displacement parameters (Å^2^)

**Table:**

	*x*	*y*	*z*	*U*_iso_*/*U*_eq_	Occ. (<1)
Pb1	0.189696 (15)	0.433942 (12)	0.821869 (11)	0.02627 (4)	
S1	0.22096 (9)	0.49032 (9)	0.11157 (7)	0.0246 (2)	
O1	0.2783 (3)	0.3880 (3)	0.0395 (2)	0.0432 (8)	
O2	0.3283 (3)	0.5673 (3)	0.1171 (3)	0.0458 (8)	
O3	0.0989 (3)	0.5773 (3)	0.0720 (3)	0.0491 (9)	
O4	−0.0031 (3)	0.1689 (3)	0.6124 (2)	0.0383 (7)	
H4	−0.0007	0.2086	0.6642	0.057*	
O5	0.2292 (3)	0.4891 (3)	0.5720 (2)	0.0382 (7)	
O6	0.1091 (3)	0.3183 (3)	0.6945 (2)	0.0364 (7)	
O7	0.4185 (4)	0.5870 (4)	0.7930 (4)	0.0693 (11)	
H7B	0.3910	0.6512	0.8307	0.104*	
H7A	0.4854	0.5373	0.8243	0.104*	
N1	0.1815 (3)	0.1900 (3)	0.9396 (2)	0.0254 (7)	
N2	0.4095 (3)	0.2831 (3)	0.7557 (3)	0.0288 (8)	
C1	0.2972 (4)	0.0991 (3)	0.9122 (3)	0.0269 (8)	
C2	0.0744 (4)	0.1459 (4)	1.0323 (3)	0.0295 (9)	
H2	−0.0063	0.2078	1.0504	0.035*	
C3	0.0764 (4)	0.0128 (4)	1.1034 (3)	0.0347 (10)	
H3	0.0005	−0.0124	1.1701	0.042*	
C4	0.1901 (5)	−0.0810 (4)	1.0751 (3)	0.0380 (10)	
H4A	0.1906	−0.1718	1.1205	0.046*	
C5	0.3070 (4)	−0.0411 (4)	0.9775 (3)	0.0306 (9)	
C6	0.4306 (5)	−0.1315 (4)	0.9431 (4)	0.0404 (11)	
H6	0.4355	−0.2236	0.9850	0.048*	
C7	0.5421 (5)	−0.0859 (4)	0.8498 (4)	0.0411 (11)	
H7	0.6222	−0.1473	0.8287	0.049*	
C8	0.5379 (4)	0.0553 (4)	0.7835 (3)	0.0313 (9)	
C9	0.6530 (4)	0.1072 (4)	0.6884 (4)	0.0376 (10)	
H9	0.7353	0.0491	0.6652	0.045*	
C10	0.6423 (4)	0.2446 (4)	0.6304 (4)	0.0368 (10)	
H10	0.7175	0.2806	0.5678	0.044*	
C11	0.5202 (4)	0.3280 (4)	0.6656 (3)	0.0346 (10)	
H11	0.5140	0.4205	0.6248	0.041*	
C12	0.4181 (4)	0.1465 (3)	0.8141 (3)	0.0272 (9)	
C13	0.1267 (4)	0.3569 (3)	0.4810 (3)	0.0231 (8)	
C14	0.0492 (4)	0.2454 (4)	0.5000 (3)	0.0262 (9)	
C15	0.0285 (4)	0.2095 (4)	0.3998 (3)	0.0342 (10)	
H15	−0.0212	0.1349	0.4117	0.041*	
C16	0.0813 (4)	0.2838 (4)	0.2825 (3)	0.0297 (9)	
H16	0.0666	0.2594	0.2158	0.036*	
C17	0.1568 (4)	0.3956 (3)	0.2634 (3)	0.0229 (8)	
C18	0.1787 (3)	0.4310 (3)	0.3625 (3)	0.0217 (8)	
H18	0.2289	0.5055	0.3499	0.026*	
C19	0.1577 (4)	0.3934 (4)	0.5883 (3)	0.0275 (9)	
O8	0.52594 (15)	1.0143 (5)	0.4767 (3)	0.0604 (17)	0.50
H8	0.4666	1.0824	0.4693	0.091*	0.50
O9	0.5369 (3)	0.8114 (4)	0.5772 (5)	0.0472 (15)	0.50
C20	0.3157 (9)	0.9480 (4)	0.6248 (6)	0.055 (2)	0.50
H20A	0.2969	0.8956	0.7083	0.082*	0.50
H20B	0.2530	0.9270	0.5829	0.082*	0.50
H20C	0.2993	1.0445	0.6231	0.082*	0.50
C21	0.4666 (8)	0.9126 (7)	0.5634 (7)	0.048 (2)	0.50

### Atomic displacement parameters (Å^2^)

**Table:**

	*U*^11^	*U*^22^	*U*^33^	*U*^12^	*U*^13^	*U*^23^
Pb1	0.03519 (7)	0.02124 (7)	0.02146 (6)	−0.00169 (5)	−0.00812 (5)	−0.00301 (5)
S1	0.0322 (4)	0.0227 (4)	0.0179 (4)	−0.0038 (3)	−0.0059 (3)	−0.0025 (3)
O1	0.0686 (19)	0.0337 (14)	0.0251 (13)	−0.0031 (14)	−0.0067 (12)	−0.0094 (11)
O2	0.0594 (16)	0.0478 (16)	0.0329 (14)	−0.0329 (13)	−0.0095 (12)	0.0017 (12)
O3	0.0382 (15)	0.0552 (18)	0.0336 (15)	0.0070 (14)	−0.0060 (12)	0.0127 (13)
O4	0.0428 (15)	0.0428 (15)	0.0243 (13)	−0.0212 (12)	−0.0008 (11)	0.0039 (11)
O5	0.0516 (15)	0.0403 (14)	0.0297 (12)	−0.0105 (12)	−0.0177 (11)	−0.0082 (11)
O6	0.0495 (15)	0.0422 (15)	0.0185 (11)	−0.0128 (12)	−0.0095 (11)	−0.0027 (10)
O7	0.069 (2)	0.0504 (19)	0.087 (3)	−0.0183 (17)	−0.0103 (19)	−0.0140 (18)
N1	0.0366 (15)	0.0219 (14)	0.0202 (13)	−0.0090 (12)	−0.0092 (11)	−0.0028 (11)
N2	0.0364 (16)	0.0230 (14)	0.0267 (14)	−0.0075 (13)	−0.0093 (12)	−0.0008 (12)
C1	0.0422 (18)	0.0198 (16)	0.0242 (16)	−0.0086 (14)	−0.0155 (14)	−0.0035 (13)
C2	0.0375 (19)	0.0298 (18)	0.0226 (16)	−0.0102 (15)	−0.0076 (14)	−0.0039 (14)
C3	0.045 (2)	0.034 (2)	0.0244 (18)	−0.0142 (17)	−0.0074 (16)	−0.0001 (15)
C4	0.058 (2)	0.0277 (19)	0.0299 (19)	−0.0206 (17)	−0.0156 (17)	0.0060 (15)
C5	0.046 (2)	0.0229 (17)	0.0282 (17)	−0.0092 (15)	−0.0196 (15)	−0.0014 (14)
C6	0.057 (2)	0.0224 (18)	0.045 (2)	−0.0028 (17)	−0.0269 (18)	−0.0005 (16)
C7	0.051 (2)	0.0279 (19)	0.049 (2)	0.0030 (18)	−0.0229 (18)	−0.0090 (17)
C8	0.0362 (18)	0.0253 (17)	0.0379 (18)	−0.0001 (15)	−0.0169 (15)	−0.0103 (14)
C9	0.0322 (19)	0.041 (2)	0.042 (2)	−0.0001 (17)	−0.0122 (16)	−0.0127 (17)
C10	0.032 (2)	0.039 (2)	0.037 (2)	−0.0086 (17)	−0.0030 (16)	−0.0074 (17)
C11	0.037 (2)	0.0315 (19)	0.0325 (19)	−0.0107 (16)	−0.0061 (16)	−0.0017 (16)
C12	0.0346 (18)	0.0212 (16)	0.0294 (17)	−0.0062 (14)	−0.0141 (14)	−0.0033 (13)
C13	0.0235 (16)	0.0239 (16)	0.0228 (15)	0.0007 (13)	−0.0084 (13)	−0.0064 (13)
C14	0.0237 (16)	0.0279 (18)	0.0247 (17)	−0.0043 (14)	−0.0042 (13)	−0.0030 (14)
C15	0.040 (2)	0.0307 (18)	0.0345 (19)	−0.0175 (16)	−0.0059 (16)	−0.0061 (15)
C16	0.0372 (19)	0.0290 (18)	0.0267 (17)	−0.0106 (15)	−0.0085 (14)	−0.0076 (14)
C17	0.0252 (16)	0.0229 (16)	0.0203 (15)	−0.0038 (14)	−0.0043 (13)	−0.0050 (13)
C18	0.0234 (16)	0.0201 (16)	0.0216 (15)	−0.0041 (13)	−0.0061 (13)	−0.0033 (12)
C19	0.0295 (18)	0.0284 (18)	0.0264 (17)	0.0026 (15)	−0.0102 (14)	−0.0095 (14)
O8	0.055 (3)	0.059 (3)	0.063 (3)	0.004 (2)	−0.014 (2)	−0.014 (2)
O9	0.042 (2)	0.051 (3)	0.051 (2)	0.024 (2)	−0.022 (2)	−0.022 (2)
C20	0.048 (3)	0.073 (4)	0.054 (3)	0.008 (3)	−0.018 (3)	−0.036 (3)
C21	0.049 (3)	0.050 (3)	0.049 (3)	−0.006 (3)	−0.018 (3)	−0.010 (3)

### Geometric parameters (Å, º)

**Table:**

Pb1—O6	2.472 (3)	C5—C6	1.414 (5)
Pb1—N2	2.473 (3)	C6—C7	1.359 (6)
Pb1—N1	2.488 (3)	C6—H6	0.9300
Pb1—O5	2.745 (3)	C7—C8	1.432 (5)
Pb1—O3^i^	2.757 (3)	C7—H7	0.9300
Pb1—O7	2.835 (4)	C8—C12	1.385 (5)
Pb1—O1^ii^	2.792 (1)	C8—C9	1.410 (5)
S1—O2	1.436 (3)	C9—C10	1.375 (6)
S1—O3	1.451 (3)	C9—H9	0.9300
S1—O1	1.454 (3)	C10—C11	1.367 (5)
S1—C17	1.772 (3)	C10—H10	0.9300
O3—Pb1^i^	2.757 (3)	C11—H11	0.9300
O4—C14	1.345 (4)	C13—C18	1.387 (4)
O4—H4	0.8200	C13—C14	1.407 (5)
O5—C19	1.239 (5)	C13—C19	1.513 (5)
O6—C19	1.280 (4)	C14—C15	1.388 (6)
O7—H7B	0.8501	C15—C16	1.379 (5)
O7—H7A	0.8500	C15—H15	0.9300
N1—C2	1.324 (4)	C16—C17	1.397 (5)
N1—C1	1.349 (4)	C16—H16	0.9300
N2—C11	1.333 (5)	C17—C18	1.378 (5)
N2—C12	1.368 (4)	C18—H18	0.9300
C1—C5	1.421 (5)	O8—C21	1.324 (7)
C1—C12	1.450 (5)	O8—H8	0.8200
C2—C3	1.381 (5)	O9—C21	1.127 (8)
C2—H2	0.9300	C20—C21	1.479 (10)
C3—C4	1.357 (6)	C20—H20A	0.9600
C3—H3	0.9300	C20—H20B	0.9600
C4—C5	1.404 (5)	C20—H20C	0.9600
C4—H4A	0.9300		
			
O6—Pb1—N2	78.58 (10)	C4—C5—C1	116.4 (3)
O6—Pb1—N1	74.87 (9)	C6—C5—C1	119.8 (3)
N2—Pb1—N1	67.00 (9)	C7—C6—C5	121.1 (3)
O6—Pb1—O5	49.83 (8)	C7—C6—H6	119.5
N2—Pb1—O5	75.83 (9)	C5—C6—H6	119.5
N1—Pb1—O5	118.07 (9)	C6—C7—C8	120.8 (4)
O6—Pb1—O3^i^	76.29 (10)	C6—C7—H7	119.6
N2—Pb1—O3^i^	140.58 (10)	C8—C7—H7	119.6
N1—Pb1—O3^i^	77.36 (9)	C12—C8—C9	117.9 (3)
O5—Pb1—O3^i^	108.70 (9)	C12—C8—C7	119.6 (3)
O6—Pb1—O7	136.69 (10)	C9—C8—C7	122.5 (3)
N2—Pb1—O7	75.24 (10)	C10—C9—C8	119.2 (4)
N1—Pb1—O7	123.15 (10)	C10—C9—H9	120.4
O5—Pb1—O7	90.23 (10)	C8—C9—H9	120.4
O3^i^—Pb1—O7	141.81 (11)	C11—C10—C9	119.4 (4)
O2—S1—O3	113.04 (19)	C11—C10—H10	120.3
O2—S1—O1	113.22 (19)	C9—C10—H10	120.3
O3—S1—O1	111.07 (19)	N2—C11—C10	123.1 (3)
O2—S1—C17	106.19 (17)	N2—C11—H11	118.4
O3—S1—C17	107.19 (16)	C10—C11—H11	118.4
O1—S1—C17	105.53 (16)	N2—C12—C8	122.1 (3)
S1—O3—Pb1^i^	128.98 (15)	N2—C12—C1	117.6 (3)
C14—O4—H4	109.5	C8—C12—C1	120.2 (3)
C19—O5—Pb1	87.7 (2)	C18—C13—C14	119.5 (3)
C19—O6—Pb1	99.6 (2)	C18—C13—C19	119.9 (3)
Pb1—O7—H7B	111.1	C14—C13—C19	120.5 (3)
Pb1—O7—H7A	111.5	O4—C14—C15	118.1 (3)
H7B—O7—H7A	105.1	O4—C14—C13	122.4 (3)
C2—N1—C1	118.1 (3)	C15—C14—C13	119.5 (3)
C2—N1—Pb1	123.9 (2)	C16—C15—C14	120.3 (4)
C1—N1—Pb1	117.9 (2)	C16—C15—H15	119.8
C11—N2—C12	118.2 (3)	C14—C15—H15	119.8
C11—N2—Pb1	123.3 (2)	C15—C16—C17	120.4 (4)
C12—N2—Pb1	118.5 (2)	C15—C16—H16	119.8
N1—C1—C5	122.7 (3)	C17—C16—H16	119.8
N1—C1—C12	119.0 (3)	C18—C17—C16	119.6 (3)
C5—C1—C12	118.3 (3)	C18—C17—S1	121.3 (3)
N1—C2—C3	123.4 (3)	C16—C17—S1	119.1 (3)
N1—C2—H2	118.3	C17—C18—C13	120.8 (3)
C3—C2—H2	118.3	C17—C18—H18	119.6
C4—C3—C2	119.3 (3)	C13—C18—H18	119.6
C4—C3—H3	120.3	O5—C19—O6	122.8 (3)
C2—C3—H3	120.3	O5—C19—C13	120.9 (3)
C3—C4—C5	120.1 (3)	O6—C19—C13	116.3 (3)
C3—C4—H4A	119.9	O9—C21—O8	115.5 (6)
C5—C4—H4A	119.9	O9—C21—C20	129.7 (5)
C4—C5—C6	123.8 (3)	O8—C21—C20	114.8 (5)
			
O2—S1—O3—Pb1^i^	−130.0 (2)	C5—C6—C7—C8	0.1 (7)
O1—S1—O3—Pb1^i^	101.4 (2)	C6—C7—C8—C12	−0.5 (7)
C17—S1—O3—Pb1^i^	−13.4 (3)	C6—C7—C8—C9	178.2 (4)
O6—Pb1—O5—C19	1.53 (19)	C12—C8—C9—C10	0.0 (6)
N2—Pb1—O5—C19	88.4 (2)	C7—C8—C9—C10	−178.8 (4)
N1—Pb1—O5—C19	34.7 (2)	C8—C9—C10—C11	−0.3 (7)
O3^i^—Pb1—O5—C19	−50.7 (2)	C12—N2—C11—C10	−1.1 (6)
O7—Pb1—O5—C19	163.1 (2)	Pb1—N2—C11—C10	179.1 (3)
N2—Pb1—O6—C19	−82.5 (2)	C9—C10—C11—N2	0.9 (7)
N1—Pb1—O6—C19	−151.5 (2)	C11—N2—C12—C8	0.8 (6)
O5—Pb1—O6—C19	−1.50 (19)	Pb1—N2—C12—C8	−179.4 (3)
O3^i^—Pb1—O6—C19	128.1 (2)	C11—N2—C12—C1	179.8 (4)
O7—Pb1—O6—C19	−28.9 (3)	Pb1—N2—C12—C1	−0.4 (4)
O6—Pb1—N1—C2	−99.5 (3)	C9—C8—C12—N2	−0.3 (6)
N2—Pb1—N1—C2	176.7 (3)	C7—C8—C12—N2	178.6 (4)
O5—Pb1—N1—C2	−125.1 (3)	C9—C8—C12—C1	−179.2 (4)
O3^i^—Pb1—N1—C2	−20.5 (3)	C7—C8—C12—C1	−0.4 (6)
O7—Pb1—N1—C2	124.2 (3)	N1—C1—C12—N2	1.4 (5)
O6—Pb1—N1—C1	84.9 (3)	C5—C1—C12—N2	−177.2 (3)
N2—Pb1—N1—C1	1.1 (3)	N1—C1—C12—C8	−179.5 (4)
O5—Pb1—N1—C1	59.2 (3)	C5—C1—C12—C8	1.8 (6)
O3^i^—Pb1—N1—C1	163.9 (3)	C18—C13—C14—O4	179.5 (3)
O7—Pb1—N1—C1	−51.5 (3)	C19—C13—C14—O4	1.5 (5)
O6—Pb1—N2—C11	101.2 (3)	C18—C13—C14—C15	1.3 (5)
N1—Pb1—N2—C11	179.5 (3)	C19—C13—C14—C15	−176.7 (3)
O5—Pb1—N2—C11	50.1 (3)	O4—C14—C15—C16	−179.3 (3)
O3^i^—Pb1—N2—C11	152.4 (3)	C13—C14—C15—C16	−1.1 (5)
O7—Pb1—N2—C11	−43.9 (3)	C14—C15—C16—C17	0.3 (6)
O6—Pb1—N2—C12	−78.6 (3)	C15—C16—C17—C18	0.2 (5)
N1—Pb1—N2—C12	−0.3 (3)	C15—C16—C17—S1	−179.1 (3)
O5—Pb1—N2—C12	−129.7 (3)	O2—S1—C17—C18	18.1 (3)
O3^i^—Pb1—N2—C12	−27.4 (3)	O3—S1—C17—C18	−103.0 (3)
O7—Pb1—N2—C12	136.3 (3)	O1—S1—C17—C18	138.5 (3)
C2—N1—C1—C5	1.0 (5)	O2—S1—C17—C16	−162.6 (3)
Pb1—N1—C1—C5	176.9 (3)	O3—S1—C17—C16	76.3 (3)
C2—N1—C1—C12	−177.6 (3)	O1—S1—C17—C16	−42.1 (3)
Pb1—N1—C1—C12	−1.7 (4)	C16—C17—C18—C13	0.0 (5)
C1—N1—C2—C3	1.2 (6)	S1—C17—C18—C13	179.4 (2)
Pb1—N1—C2—C3	−174.4 (3)	C14—C13—C18—C17	−0.8 (5)
N1—C2—C3—C4	−3.1 (6)	C19—C13—C18—C17	177.2 (3)
C2—C3—C4—C5	2.6 (6)	Pb1—O5—C19—O6	−2.7 (3)
C3—C4—C5—C6	179.0 (4)	Pb1—O5—C19—C13	179.1 (3)
C3—C4—C5—C1	−0.5 (6)	Pb1—O6—C19—O5	3.0 (4)
N1—C1—C5—C4	−1.3 (6)	Pb1—O6—C19—C13	−178.7 (2)
C12—C1—C5—C4	177.3 (4)	C18—C13—C19—O5	0.0 (5)
N1—C1—C5—C6	179.1 (4)	C14—C13—C19—O5	178.0 (3)
C12—C1—C5—C6	−2.2 (6)	C18—C13—C19—O6	−178.3 (3)
C4—C5—C6—C7	−178.1 (4)	C14—C13—C19—O6	−0.4 (5)
C1—C5—C6—C7	1.3 (6)		

Symmetry codes: (i) −*x*, −*y*+1, −*z*+1; (ii) *x*, *y*, *z*+1.

### Hydrogen-bond geometry (Å, º)

**Table:**

*D*—H···*A*	*D*—H	H···*A*	*D*···*A*	*D*—H···*A*
O4—H4···O6	0.82	1.83	2.522 (4)	141
O7—H7*A*···O2^iii^	0.85	2.11	2.959 (5)	172

Symmetry code: (iii) −*x*+1, −*y*+1, −*z*+1.

## References

[bb1] Brandenburg, K. (2006). *DIAMOND* Crystal Impact GbR, Bonn, Germany.

[bb2] Chen, Z.-F., Shi, S.-M., Hu, R.-X., Zhang, M., Liang, H. & Zhou, Z.-Y. (2003). *Chin. J. Chem.* **21**, 1059–1065.

[bb3] Du, Z.-X., Han, M.-L., Wang, J.-G. & Qin, J.-H. (2006). *Acta Cryst.* E**62**, m3047–m3048.

[bb4] Fan, S.-R. & Zhu, L.-G. (2005*a*). *Acta Cryst.* E**61**, m1298–m1300.

[bb5] Fan, S.-R. & Zhu, L.-G. (2005*b*). *Acta Cryst.* E**61**, m1598–m1600.

[bb6] Fan, S.-R. & Zhu, L.-G. (2006). *Inorg. Chem.* **45**, 7935–7942. 10.1021/ic060871v 16961387

[bb7] Farrugia, L. J. (1997). *J. Appl. Cryst.* **30**, 565.

[bb8] Li, J.-F., Zhao, Y.-J., Li, X.-H. & Hu, M.-L. (2004). *Acta Cryst.* E**60**, m1210–m1212.

[bb9] Oxford Diffraction (2011). *CrysAlis PRO* Oxford Diffraction Ltd, Yarnton, Oxfordshire, England.

[bb10] Sheldrick, G. M. (2008). *Acta Cryst.* A**64**, 112–122. 10.1107/S0108767307043930 18156677

[bb11] Westrip, S. P. (2010). *J. Appl. Cryst.* **43**, 920–925.

